# Identification of protein kinase G I alpha interacting proteins as potential targets to prevent cardiac remodeling

**DOI:** 10.1186/2050-6511-14-S1-P10

**Published:** 2013-08-29

**Authors:** Robert Blanton, Angela Lane, Mark Aronovitz, Guang-Rong Wang, Robrecht Thoonen, Roger Davis, Michael Mendelsohn, David Kass, Richard Karas

**Affiliations:** 1 Molecular Cardiology Research Institute, Tufts Medical Center, Boston, MA, USA; 2 Department of Biochemistry, University of Massachusetts School of Medicine, Worcester, MA, USA; 3 Merck Pharmaceuticals, Rahway, NJ, USA; 4 Department of Cardiology, Johns Hopkins Medical Institutions, Baltimore, MD, USA

## Background

We recently reported that mutation of the cGMP-dependent Protein Kinase G I alpha (PKGIα) N-terminal leucine zipper (LZ) domain (in the PKGIα LZ mutant, or LZM, mouse) accelerates cardiac remodeling and heart failure after left ventricular (LV) pressure overload, and prevents the anti-remodeling effect of sildenafil [1]. We therefore hypothesized that PKGIα attenuates remodeling by regulating cardiac signaling pathways that are dependent on substrate interactions mediated by its LZ domain. As a first step to identifying cardiac proteins downstream of PKGIα, we screened myocardial lysates for PKGIα LZ domain-interacting proteins. Our previous work revealed a requirement for the PKGIα LZ domain for the activation of anti-remodeling myocardial JNK activity after LV pressure overload. MLK3 is a MAPKKK that contains an LZ domain and activates JNK.

## Results

We now demonstrate, by immunoprecipitation, that MLK 3 interacts with the PKGIα LZ domain in myocardial lysates. We show further that 8-Br-cGMP induces MLK3 phosphorylation on Thr277 and Ser281 in WT, but not LZM lysates. And, in 293 cells transfected with FLAG-MLK3, 8Br-cGMP induced PKGIα-MLK3 co-precipitation, and increased MLK3 phosphorylation on Thr277/Ser281. Co-transfection of MLK3 and PKGIα also induced MLK3 phosphorylation. We next examined the cardiovascular effect of MLK3 deletion in vivo. Male 8 week old MLK3-/- mice display basal bi-ventricular hypertrophy compared with littermate controls (LV/Tibia length 42.8 ± 0.6 mg/cm in WT, 52.9 ± 1.8 in MLK3 -/-; *P*<0.01; RV/TL 10.8 ± 0.1 mg/cm in WT, 13.3 ± 0.3 in MLK3-/-; *P*<0.01; n= 7 WT, 5 MLK3-/-). By 14-16 weeks of age, LVH progressed in the MLK3-/- mice (LV/TL 47.7 ± 1.3 mg/cm in WT, 59.8 ± 7.5 in MLK3-/-; n= 6 WT, 9 MLK3-.-; *P*<0.01). Arterial blood pressure was modestly increased, though still normal, in MLK3-/- mice (SBP 93 ± 1 in WT, 113 ± 1 in MLK3-/-). And, 14-16 week MLK3-/- mice have impaired LV diastolic function (tau 3.2 ± 0.1 ms WT, 3.7 ± 0.1 MLK3-/-; *P* 0.06).

## Conclusion

Our studies reveal a novel function of MLK3 as a myocardial PKGIα effector and inhibitor of LVH. These results support the strategy of exploring LZ-dependent PKGIα substrates in the myocardium to identify potential therapeutic targets for cardiac remodeling.
